# Tumor-Induced CD8+ T-Cell Dysfunction in Lung Cancer Patients

**DOI:** 10.1155/2012/741741

**Published:** 2012-10-17

**Authors:** Heriberto Prado-Garcia, Susana Romero-Garcia, Dolores Aguilar-Cazares, Manuel Meneses-Flores, Jose Sullivan Lopez-Gonzalez

**Affiliations:** Departamento de Enfermedades Cronico-Degenerativas, Instituto Nacional de Enfermedades Respiratorias Ismael Cosío Villegas, Calzada de Tlalpan 4502, Seccion XVI, 14080 Mexico City, Mexico

## Abstract

Lung cancer is the leading cause of cancer deaths worldwide and one of the most common types of cancers. The limited success of chemotherapy and radiotherapy regimes have highlighted the need to develop new therapies like antitumor immunotherapy. CD8+ T-cells represent a major arm of the cell-mediated anti-tumor response and a promising target for developing T-cell-based immunotherapies against lung cancer. Lung tumors, however, have been considered to possess poor immunogenicity; even so, lung tumor-specific CD8+ T-cell clones can be established that possess cytotoxicity against autologous tumor cells. This paper will focus on the alterations induced in CD8+ T-cells by lung cancer. Although memory CD8+ T-cells infiltrate lung tumors, in both tumor-infiltrating lymphocytes (TILs) and malignant pleural effusions, these cells are dysfunctional and the effector subset is reduced. We propose that chronic presence of lung tumors induces dysfunctions in CD8+ T-cells and sensitizes them to activation-induced cell death, which may be associated with the poor clinical responses observed in immunotherapeutic trials. Getting a deeper knowledge of the evasion mechanisms lung cancer induce in CD8+ T-cells should lead to further understanding of lung cancer biology, overcome tumor evasion mechanisms, and design improved immunotherapeutic treatments for lung cancer.

## 1. Introduction 

Lung cancer is the leading cause of cancer-related mortality in developed countries and the second leading cause of death in countries with emerging economies. Worldwide, lung cancer is one of the most commonly diagnosed neoplasias, representing 13% of all cancer cases and approximately 18% of all cancer deaths [1–3]. In countries with emerging economies, the adoption of cancer-associated lifestyles such as reduced physical activity, smoking, unhealthy dietary habits, the increased air pollution, and an aging population has led to a boost in the prevalence of lung cancer [1, 2, 4].

Lung carcinomas are classified as either of two types: small cell lung carcinoma (SCLC) and non-SCLC (NSCLC). NSCLC accounts for approximately 85% of all lung cancer cases and includes three histological subtypes: squamous cell carcinoma, adenocarcinoma, and large cell carcinoma. Treatment of NSCLC involves surgery in the early stages, chemotherapy with concurrent radiation for some locally advanced cancers, and palliative chemotherapy for metastatic disease. In developing countries, most lung cancer diagnoses are performed at advanced stages of lung malignancy and therefore 5-year survival rates remain low [4, 5].

The limited success of chemotherapy has emphasized the need to develop new therapeutic strategies such as immunotherapy. However, the multifaceted nature of the immune escape mechanisms of lung tumor cells is a major obstacle to the potential application of immunotherapy in lung cancer patients. There is a need, therefore, to elucidate and characterize these immune escape mechanisms to develop strategies to counteract them, thus enhancing the efficacy of T-cell-based immunotherapies.

Several tumor evasion mechanisms to immune responses have been reported [6]; however, few have been shown to participate in lung cancer. In a recent paper, Ding and Zhou [7] described the role of CD4+ T-cells and their subsets in tumor immunity. In this review, we focus on the alterations induced by lung tumors on CD8+ T-cells. 

## 2. Chronic Inflammation ****and Immunosuppression in ****Lung Cancer

 Chronic inflammation has been associated with increased risk of tumor development and progression. Tumor promoting factors, such as protein and DNA damage through oxidative stress, as well as, angiogenesis and tissue remodeling, are induced by chronic inflammation. Substances such as asbestos, cigarette smoke, and wood smoke are known to cause a chronic inflammatory state, which in turn promotes tumorigenesis [8]. Also, pulmonary disorders such as chronic obstructive pulmonary disease (COPD)/emphysema and pulmonary fibrosis, which are associated with greater risk for developing lung cancer, are characterized by abundant and deregulated inflammation [9, 10]. 

Cancer induces non-MHC-restricted inflammatory responses in the host, as in most chronic diseases; both the innate and adaptive components of the immune response play a role in the control of tumor growth and metastasis [8]. However, tumors impair the inflammatory responses and take advantage of the responses to promote tumor survival, proliferation, and metastasis. Therefore, the presence of leukocytes within a tumor may be a consequence of an inflammatory reaction that supports either the spread of tumor cells or the protective host antitumor immune responses [11]. The immunoediting theory has been proposed to explain the interaction between tumor cells and the immune system. This theory involves three phases: elimination, equilibrium, and escape [12, 13].

Lung tumors have been considered poorly immunogenic and incapable of inducing an immune response. One factor that may contribute to this low responsiveness is smoking, which is well known for increasing the risk of developing lung cancer. Smoking has been shown to exert several proinflammatory effects on immunity [14]. For example, smoking increases production of several proinflammatory cytokines such as Tumor Necrosis Factor-alpha (TNF-*α*), Interleukin 1 (IL-1), IL-6, and IL-8 and decreases anti-inflammatory cytokines, such as IL-10. Proinflammatory cytokines IL-6 and TNF-*α* are associated with chronic inflammation and immunosuppression [8].

Dendritic cell (DC) maturation is inhibited by cigarette smoking, as demonstrated by reduced cell surface expression of MHC class II and the costimulatory molecules CD80 and CD86. Consequently, DCs from cigarette smoke-exposed animals show reduced capacity to stimulate and activate antigen-specific T-cells *in vitro*; this phenomenon is consistent with a reduced antigen-specific T-cell proliferation in smoke-exposed mice observed *in vivo* [15]. Smoke-induced defects in DC function may lead to impaired T-cell function and inhibit tumor immunosurveillance [14, 15]. Moreover, asbestos, which is another factor associated to increased risk for developing tumors such as mesothelioma and lung cancer, has been reported to promote reduction of antitumor immunity. Asbestos reduces interferon-gamma (IFN-*γ*) production in stimulated CD4+ T-cells *in vitro*; also, asbestos reduces the expression of chemokine receptors such as CXCR3, which is expressed by memory T-cells and macrophages [16]. IFN-*γ* induces CXCR3 ligands expression among which the chemokine CXCL10 has been shown to inhibit NSCLC tumorigenesis and spontaneous metastases [17].

However, there is circumstantial evidence that immunosuppression is a risk factor for developing lung cancer. Within the population of HIV-positive patients the incidence of lung cancer has been estimated to be 2 to 4 times higher with respect to that of the general population. Several factors, including viral load, CD4+ T-cell count, immunosuppression, and smoking, have been linked to development of lung cancer in these patients [18–20]. Moreover, tobacco and immunosuppression are risk factors for developing lung cancer after liver transplantation [21]. In another study by Engels et al. [22], lung cancer risk, among other carcinomas such as liver and kidney carcinomas, was reported to be higher in recipients receiving solid organ transplantation. Taken together these reports suggest that the immune system might control to some extent development of lung tumors.

## 3. Lung Cancer and Pleural Effusion

A frequent inflammatory condition present in lung cancer, particularly in lung adenocarcinomas, is the formation of pleural effusions as a consequence of tumor invasion of the pleura in late stages of cancer. Liquid accumulation in this compartment leads to the formation of a pleural effusion, which occurs in 15%–20% of primary lung cancer cases. In these patients, the pleural effusion is detected as an amount of fluid varying between 300 and 1500 mL. Appearance of pleural effusion is an ominous prognostic sign for lung cancer patients, because the presence of this condition is associated with a poor prognosis with a median survival of 4 months [23].

Cytology studies of pleural effusions indicate that most of these effusions contain high proportions of both neoplastic and inflammatory cells [24, 25], which makes this biological material a suitable model for studying the host immune system and malignant cell interactions [24–28]. Tissue samples collected by biopsies limit the amount of material obtained, and therefore it is not possible to perform concurrent phenotyping, quantification, and functionality studies on distinct immune cells in tumor-infiltrating lymphocytes (TILs) from the same tissue sample [29]. Conversely, the *ex vivo* model using immune cells from malignant effusions allows to study the effects of tumor cell-mediated alterations in T-cells. Data obtained from the study of malignant effusion provide a comprehensive and integral vision of the effects of tumor cell-mediated alterations in distinct subpopulations of T-cells and particularly in CD8+ T-cells.

## 4. Tumor-Associated Antigens in Lung Cancer 

During oncogenesis, transformed cells gradually acquire mutations and epigenetic alterations that increase the quantity of antigens expressed in normal tissue, mutated proteins, novel epitopes encoded within alternative open reading frames, intronic sequences, or products that result from protein splicing. These antigens are collectively designated as tumor antigens. Tumor-associated antigens were initially characterized in melanoma by analyzing TILs [30, 31]. The identification of tumor-associated antigens has enabled the development of vaccines that induce a potent antigen-specific CD8+ T-cell response against tumors [30, 32]. 

The consensus is that tumors express at least five types of antigens that can be recognized by the immune system: (a) antigens coded by oncogenic viruses. (b) MHC-restricted tumor-associated peptides shared by histologically distinct tumors and silent in normal tissues, except for germ cells in the ovaries and testes [30, 31]. In lung cancer, melanoma-specific antigen A3 (MAGE A3) is one of the best characterized [33]. (c) Overexpressed antigens such as survivin [34] and Wilms' tumor gene WT1 product are some of the few identified in lung tumors [31, 35, 36]. (d) Differentiation-specific antigens such as melanoma- and melanocyte-associated tyrosinase-1. In lung cancer, differentiation antigens have not been identified so far [31]. (e) Unique antigens are generated by point mutations in ubiquitously expressed genes and regulate key cellular functions. Some mutated antigens reported in lung cancer are p53 [37], the elongation factor 2 gene [38], actinin-4 [39], malic enzyme [40], and NF-YC [31, 41].

Melanoma has been considered an “immunogenic” tumor due to the presence of antitumor immune cells within the tumor tissue, the identification of tumor antigens that are capable of being recognized by the host [31, 42], and the clinical benefits reported with the application of some immunotherapeutic schemes [32]. In contrast, lung cancers show a low infiltration of TILs. Qualitative and quantitative abnormalities in the distinct immune cells infiltrating lung tumors, and particularly in CD8+ T-cells, have also been reported [43–46]. One of the most common evasion mechanisms against CD8+ T-cells in cancer is loss or downregulation of HLA molecules expression. Lung cancer cells have been shown to downregulate HLA I expression which may lead to develop cancer [47, 48]. Also, the application of lung tumor antigens have had limited success as antitumoral vaccines against lung cancer [49, 50]. For these reasons, lung tumors have been considered poorly immunogenic. However, lung tumors express tumor associated antigens, which can be recognized by the CD8+ T-cells of the host immune system. Several studies have reported that cytotoxic T lymphocyte clones can be established; these clones are MHC class I-restricted and show specific cytotoxicity against autologous target cells [51–55]. Thus, the poor immune response observed against lung cancer may be attributed to the evasion mechanisms presented by lung tumor cells.

## 5. CD8+ T-Cells Infiltrating Lung Tumors

CD8+ T-cells are a crucial component of the cellular immune response, which is necessary for the control of a variety of bacterial and viral infections. These cells also represent a major arm of the cell-mediated antitumor immune response [56]. CD8+ T-cell protection is mediated by its ability to specifically target host cells compromised by microbial infection or oncogenic transformation. Following exposure to antigens by DCs in an appropriate inflammatory environment, CD8+ T-cells undergo a period of massive expansion, activation, and differentiation to terminally differentiated cells with effector functions. Once the pathogenic process is resolved, most effector CD8+ T-cells undergo apoptosis, leaving a long-lived subset of memory cells. These cells possess an enhanced ability to control secondary exposures to antigens [32, 57, 58], which is attributed to their increased frequency, rapid acquisition of effector functions, and recruitment to the tumor sites. In both animal models and humans, CD8+ T-cells have been shown to play an important role in the host's defense against malignancies [59]. Therefore, most cancer vaccine strategies have focused on the induction of effector CD8+ T-cells that kill tumor cells [30, 32]. 

TILs have been shown to contribute to the clinical outcome of human cancers. A high infiltration of T-cells is associated with good clinical outcome in many different kinds of cancers. In tumors such as colorectal cancer [60, 61] and ovarian carcinoma [62], high densities of intratumoral memory (CD45RO+) cells and CD8+ T-cells are localized and correlate with favorable prognosis. 

Accordingly, some reports show that the presence of TILs with memory phenotype in lung cancer is predictive of a favorable clinical outcome [44, 46, 63, 64]. Ruffini et al. [65] showed that CD8+ T-cells were associated with prolonged survival in lung cancer; this association was only found in squamous cell carcinomas. In another study, CD8+ T-cell infiltrations were observed predominately along the invasive margin (peritumoral distribution) as well as within lymphoid aggregates that are formed at adjacent stromal tissue, which have been termed as tertiary lymphoid structures [44]. These tertiary lymphoid structures are associated with long-term survival in lung cancer patients [44]. 

Interestingly, Wakabayashi et al. [43] showed that high numbers of CD4+ T-cells, but not CD8+ T-cells, within cancer cell nests are positively correlated with favorable prognosis in lung cancer patients. This finding suggests that CD4+ T-cells may be required for initiating and maintaining antitumor immune responses; given that, without CD4+ T-cell help, the resultant CD4-unhelped CD8+ T-cells do not differentiate into sustainable memory cells [57]. Hiraoka et al. [66] found a synergistic effect of simultaneous high CD4+ and CD8+ T-cell infiltrations in cancer stroma, from resected tumor specimens, as a favorable prognostic factor in lung cancer patients. Nevertheless, more recently, Al-Shibli et al. [64] showed that high densities of CD8+ T-cells in the stroma significantly correlated with an improved survival in patients with NSCLC (stages I to IIIa). This correlation was found to be independent of infiltrating CD4+ T-cells. Even though the role of infiltrating CD4+ T-cells as an independent factor predicting favorable outcome is still controversial, these studies suggest that low infiltration of CD8+ T-cells is associated with a poor clinical outcome in lung cancer patients. 

A similar behavior has been found in the pleural compartments of lung cancer patients. Our group and other authors have reported that the CD8+ T-cell subpopulation in pleural effusion is reduced, while the CD4+ T-cell subpopulation is increased [24, 27, 67].

The phenotyping of pleural effusion CD8+ T-cells from lung cancer patients shows an elevated population of memory (CD45RA−CD45RO+CD27+CD28+) CD8+ T-cells and a low proportion of terminally differentiated (CD45RA+CD45RO−CD27−CD28−) CD8+ T-cells, which is similar to data from TILs. The selective recruitment of memory CD8+ T-cells in lung tumors may be related to the presence of chemotactic factors for this subset (e.g., CCL21, CCL5 and CCL2), as has been reported by de Chaisemartin et al. [68].

Even though memory CD8+ T-cells infiltrate lung tumors, both in TIL and malignant effusions, the CD8+ T-cells are functionally impaired and are poorly responsive or unresponsive to several T-cell-activating stimuli. CD8+ T-cells have reduced proliferation rate, diminished production of some Th1 cytokines, and reduced cytotoxic potential [24, 45, 69]. These findings suggest that CD8+ T-cells, located in contact or in proximity to the tumor, are profoundly affected by tumor-derived factors compared with those CD8+ T-cells located in sites that are more distant from the tumor. 

CD8+ T-cells from both TILs and pleural effusions share a similar pattern of dysfunctions. Due to the advanced stage of lung cancer, the evasion mechanisms of lung tumors may be comparable at the local level (tumor *in situ*) and at the pleural compartment (metastatic tumor). Thus, the study of malignant effusions may provide future insight into the interaction between tumor cells and immune cells.

**Figure 1:**
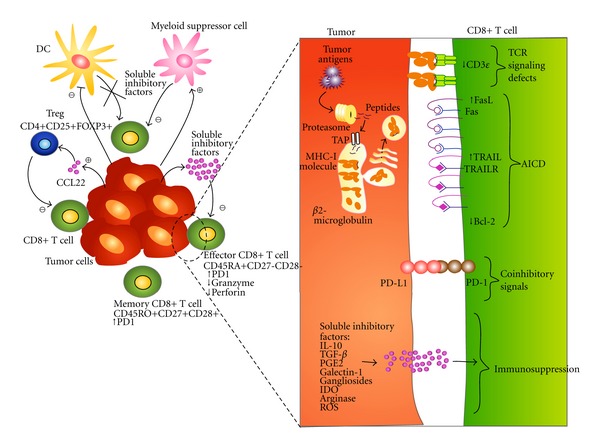
Alterations induced in the CD8+ T-cells by lung cancer. Tumor, stroma, cells, and soluble factors in the microenvironment inhibit DCs recruitment and induce the presence of immune cells with suppressor activity, such as myeloid suppressor cells and Tregs, which results in (1) blocking the differentiation program of CD8+ T-cells keeping them in a memory stage and diminishing the terminally-differentiated CD8+ T-cell subset, (2) reduced expression of cytolytic molecules granzymes and perforin, (3) reduced expression of CD3*ε*, altering the signaling pathway through TCR ligation, (4) PD-L1/PD-1 interaction that induces on CD8+ T-cell decreased TCR-mediated proliferation and cytokines production, (5) sensitization of CD8+ T-cell to apoptosis mediated by AICD.

Effector CD8+ T-cells should be present at the tumor sites eliminating the lung tumor cells. Their absence may be because tumor cells block the differentiation process from memory cells to terminally differentiated CD8+ T-cells. This phenomenon may be mediated by the following; see Figure 1.

- Immunosuppression factors in the microenvironment. Lung cancer cell lines release immunoregulatory cytokines such as IL-10 and Transforming Growth Factor beta (TGF-*β*) [70, 71] as well as enzymes that catabolize amino acids that are important for T-cell effector functions (e.g., indoleamine-2,3-dioxygenase, IDO, and arginase) [6, 72].
- Deficient tumor antigen presentation by DCs due to the absence of tumor-infiltrating DCs or to the segregation of DCs, which hinders their migration to and maturation in the lymphatic node, thereby blocking induction of an antitumor immune response.
- Reduced production of cytokines by helper CD4+ T-cells for costimulation of CD8+ T-cells, such as IL-15 [73, 74].
- Suppression induced by lung tumors through the recruitment of regulatory T-cells (Tregs) or other regulatory populations, such as myeloid suppressor cells. Tregs play an active role in establishing and maintaining immunological unresponsiveness to self-constituents and negative control of various immune responses to nonself-antigens. Tregs, identified as CD4+CD25, CD4+CD25+FOXP3+ or CD4+CD25+CD127− T-cells, have been found in pleural effusions as well as in TILs from lung cancer patients [75–79]. Recently, tumor infiltrating FOXP3+ cells have been associated with low overall and relapse-free survival in NSCLC [78]. Nevertheless, studies which associate CD8+ T-cell with Tregs infiltration in lung cancer have not been done so far.
- Downregulation of CD3*ε* expression in memory CD8+ T-cells. A deficiency in CD3*ε* has been reported by our group in pleural effusion CD8+ T-cells from lung cancer patients [80]. CD3*ε* is an essential component of the CD3 complex, which is responsible for translating the TCR signaling. The reduced expression of the CD3*ε* chain may block the terminal-differentiation program of CD8+ T-cells. Accordingly, T-cells isolated from the lung tumor microenvironment are nonresponsive to triggering through the TCR, which can be reversed by IL-12 [81]. However, IL-12 administered *in situ* in murine lung tumors induces T-cell death [82].

An efficient generation of effector CD8+ T-cells ideally leads to clearance of the tumor cells. In a variety of infections in mouse and primates, pathogens have been shown to escape immune control and become persistent through the long-term antigenic stimulation of responding CD8+ T-cells. Chronic stimulation results in a progressive loss of the effector function of CD8+ T-cells and the expression of markers associated with T-cell exhaustion, such as the PD-1 coinhibitory molecule [83–85]. In addition, chronic stimulation has been shown to sensitize CD8+ T-cells to activation-induced cell death (AICD) [85]. A similar phenomenon has been observed in some tumors; for example, in melanoma, CD8+ T-cells specific for MART-1 express higher levels of PD-1 and reduced levels of IL-2 and IFN-*γ* [86]. In mouse tumor models, the blockade of PD-L1 leads to increased expansion of tumor-specific T-cells and decreased numbers of apoptotic T-cells [83]. Pleural effusion CD8+ T-cells from lung cancer patients express cell markers associated with a memory-like phenotype (CD45RA−CD45RO+CD27+Granzyme A^low^Perforin−), similar to those markers found in CD8+ T-cells from chronic viral infections, which suggests that CD8+ T-cells may be exhausted [85]. Recently, Zhang reported that CD8+ T-cells from TILs of NSCLC showed increased expression of PD-1 [87]. Thus, the continued presence of the tumor may sensitize memory CD8+ T-cells to AICD before they reach the effector stage. However, the evaluation of exhaustion in lung tumor-specific CD8+ T-cells has not been possible because lung tumor-associated antigens are not expressed in all cancer patients. Nevertheless, clinical trials using PD-1-blocking antibodies are underway for NSCLC [88].

## 6. CD8+ T-Cells Are Impaired in ****Their Lytic Function

The perforin-granzyme (or granule exocytosis) pathway is the classic effector mechanism that CD8+ T-cells and NK cells use to lyse target cells. This pathway is responsible for the elimination of intracellular bacterial and viral infections and for tumor cell destruction. Perforin and granzymes are highly expressed in terminally differentiated CD8+ T-cells [56].

Reduced numbers of perforin- and granzyme-positive cells have been observed in TILs from lung cancer patients using immunohistochemistry methods, suggesting that TILs are dysfunctional [89]. However, memory (CD45RO+) CD8+ T-cells express low levels of perforin [90, 91]; thus the low expression of this molecule in TILs may reflect the fact that terminally differentiated cells infiltrating tumor cells are present in low proportions. Nevertheless, the impaired expression of perforin compared with granzyme A has been found by our group in terminally differentiated CD8+ T-cells from malignant pleural effusion and to a lesser extent in the corresponding CD8+ T-cell subset from peripheral blood of lung cancer patients [24]. 

Cytolytic activity from both TILs and pleural effusion lymphocytes has been determined using autologous tumor cells. TILs from lung cancer patients present poor cytolytic activity against autologous tumor cells, which is recovered after treating T-cells with recombinant IL-2 [46]. Also, pleural effusion T-cells from lung cancer patients have been shown to exhibit poor cytolysis against autologous tumor, Daudi and K562 cells. Similar to TILs, stimulation with anti-CD3 and IL-2 restored cytotoxicity against tumor cells [28, 92]. IL-15 induces the proliferation of memory CD8+ T-cells independently of antigen and increases their effector function [93]; accordingly, malignant pleural effusion T-cells treated with IL-15 exert cytolysis against autologous tumor cells [69]. Combinations of other stimuli such as IL-2 plus IL-7, IL-2 plus IL-12, or IL-2 plus TCR-CD3 engagement also reverse the immunosuppressed state of pleural effusion T-cells; remarkably, cytolysis of autologous tumor cells is mainly mediated by CD8+ T-cells [94]. 

These data support the conclusion that, in lung cancer, terminally differentiated CD8+ T-cells have a defective cytolytic function, which can be restored by using a combination of cytokines and TCR-engagement stimuli. Chronic stimulation results in the loss of effector CD8+ T-cell function; perforin and granzyme expressions are downregulated in viral infections [83]. Similar alterations in CD8+ T-cells may be associated with chronic stimulation of immune cells by lung tumors.

## 7. CD8+ T-Cell Death in Lung Cancer: The Role of AICD

Numerous studies have demonstrated that a high frequency of T-cell apoptosis occurs in several types of cancer; in particular, CD8+ T-cells are more susceptible to apoptosis [95–97]. Remarkably, T-cell death is not limited to the tumor site because increased apoptosis has been found in T-cells from peripheral blood of patients with head and neck carcinomas, breast carcinomas, or melanomas [95–97]. In lung cancer, cell death of the effector CD8+ T-cell subset may be responsible for its reduced presence; given that a high percentage of pleural effusion and peripheral blood CD8+ T-cells express Fas. This phenomenon may lead to the apoptosis of Fas-expressing CD8+ T-cells when they reach the terminally differentiated stage. 

We recently found that peripheral blood CD4+ and CD8+ T-cells from lung cancer patients show a high susceptibility to spontaneous apoptosis compared with T-cell subpopulations from healthy donors. This phenomenon is mainly observed in CD8+ T-cells. Nevertheless, susceptibility to spontaneous apoptosis does not lead to a reduction of the CD8+ T-cell subpopulation in peripheral blood [24, 98].

The Fas/Fas ligand (FasL) pathway has been proposed to be responsible for the spontaneous apoptosis observed in T-cells. Peripheral blood CD8+ T-cells from cancer patients increase apoptosis after the engagement of the Fas receptor [95, 99, 100]. However, no apoptosis of CD4+ and CD8+ T-cells from lung cancer patients was observed after treatment with agonistic anti-Fas antibodies [101]. 

The binding of the Fas ligand (FasL) on the tumor cell to the Fas receptor on the T-cell, a hypothesis known as tumor counterattack, has been suggested as responsible for T-cell death [100]. Though controversial, several reports have shown that a variety of human tumors express and secrete functional FasL (contained in microvesicles). However, other authors and our group have reported that lung cancer cells do not express FasL. This has been demonstrated in lung cancer cell lines, tumors cells from pleural effusions, and resected tumor tissue [80, 102]. Thus, spontaneous apoptosis in lung cancer is not mediated by the Fas receptor; other death receptors (TNFR1, DR4, DR5, etc.), however, may induce this phenomenon. In addition, spontaneous apoptosis may be the consequence of other factors (described below) that are systemically released by tumor or stromal cells. 

Tumor cells release tumor antigens that chronically stimulate CD8+ T-cells [103, 104]. This chronic stimulation may sensitize CD8+ T-cells from lung cancer patients to AICD as has been shown in TILs from various types of human [55, 105, 106] and murine tumors [107, 108]. Chemokines, cytokines or other soluble factors secreted by lung tumors or stromal cells may also induce and amplify non-HLA restricted inflammatory responses, leading to an increased susceptibility to AICD in CD8+ T-cells. 

**Figure 2:**
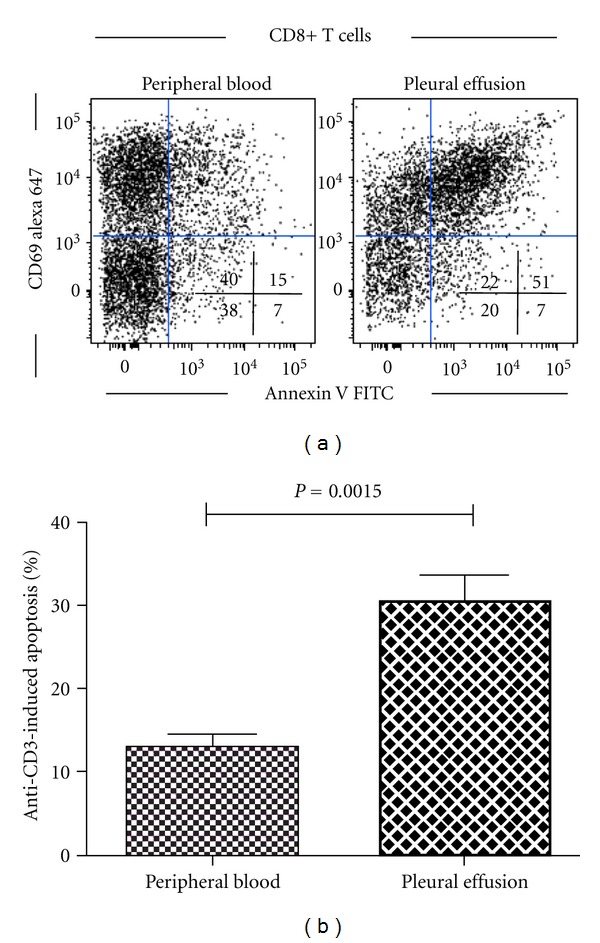
Pleural effusion, but not peripheral blood, CD8+ T-cells from lung cancer patients are sensitive to AICD. Both pleural effusion and peripheral blood CD8+ T-cells from lung cancer patients (*n* = 10) were stimulated with anti-CD3 mAbs for 24 h; then apoptosis was determined by annexin V binding in activated CD8+CD69+ T-cells by multiparametric flow cytometry; propidium iodide was used to exclude necrotic cells. (a) Results from a lung cancer patient. (b) Anti-CD3 induced apoptosis was determined as described in [101], comparison was made by paired Student's *t*-test. Bars depict the mean ± Standard Error.

Accordingly, we recently reported that CD8+, but not CD4+, T-cells from malignant pleural effusions undergo AICD and this phenomenon is not observed in peripheral blood CD4+ and CD8+ T-cells [101] (see Figure 2). We found that AICD is associated with the upregulation of FasL and TRAIL expression and reduced the expression of the antiapoptotic molecule Bcl-2 [101]. FasL expression has also been found in TILs from lung tumors [102]. AICD is preferentially observed in memory and terminally differentiated CD8+ T-cells. In contrast, memory CD8+ T-cells from healthy donors have been shown to be resistant to AICD [109, 110].

Polyclonal stimulation of pleural CD8+ T-cells leads to AICD, a phenomenon that potentially involves both tumor- and nontumor-specific CD8+ T-cells. Kilinc et al. [82], in a murine lung tumor model, observed that intratumoral delivery of IL-12 results in the activation and subsequent death of total effector/memory CD8+ T-cells *in situ*. The factors that can contribute to the increased susceptibility of pleural effusion CD8+ T-cells to AICD are the following: (a) gangliosides released by lung tumor or stromal cells, given that these molecules sensitize activated T-cells to apoptosis *in vitro* [111, 112]; (b) diminished levels of CD3*ε* in CD8+ T-cells may lead to the dysfunction of CD8+ T-cell responses and enhanced T-cell apoptosis [80]; (c) the chronic presence of tumor antigens and damage associated molecular patterns (DAMPs) secreted by tumor or stromal cells in the pleural compartment. DAMPs have been shown to induce a high production of reactive oxygen and nitrogen species, which damage the memory or terminally differentiated CD8+ T-cells [113–115].

Therefore, the susceptibility to AICD of malignant effusion-derived memory CD8+ T-cells may prevent these cells from becoming terminally differentiated. In a similar way, nonantigen-specific CD8+ T-cells become susceptible, in a bystander fashion, to AICD after TCR stimulation in some viral infections [116]. Bystander sensitization to AICD has been proposed as a mechanism for immune deficiencies associated with persistent viral infections involving chronic T-cell responses [116]. In lung cancer patients, a similar but deregulated phenomenon may explain the susceptibility to AICD of CD8+ T-cells in the pleural compartment [24]. 

## 8. Concluding Remarks

Several clinical trials have been conducted using intrapleural administration of diverse combinations of biological response modifiers to increase the host immune response against tumor cells [117–119]. However, these treatments have failed to show significant clinical benefits in terms of patient survival or quality of life. Another approach in immunotherapeutic treatment is adoptive cell transfer, in which immune cells from cancer patients are expanded *ex vivo*. These cells are then infused back into the patient with the hope that they reach the tumor and induce tumor cell death. This approach has been attempted in a number of clinical trials for lung cancer with little success [120, 121]. The susceptibility to AICD of CD8+ T-cells may explain the limited success of these therapies; thus, blocking the apoptotic loop is essential for the success of T-cell-based immunotherapeutic regimens for patients with lung cancer.

The role that CD8+ T-cells plays against tumor cells is crucial. However, based on the information presented above, lung tumor cells induce on CD8+ T-cells a series of quantitative and qualitative alterations that hamper their full participation in tumor recognition and destruction (see Figure 1). As similar alterations are found both in primary as in metastatic tumors (pleural effusions), the amount of tumor and immune cells interacting in this anatomical compartment allow a more integral and complete study of the several evasion mechanism shown by lung tumor cells. 

Getting a deeper knowledge of the evasion mechanisms that lung cancer induces in the cells of the immune system, and particularly in CD8+ T-cells, should lead to further understanding of lung cancer biology, downregulation of evasion mechanisms due to tumor cells and design of improved immunotherapeutic CD8+ T-cell-based regimes.
